# Effects of a nurse-coordinated prevention programme on health-related quality of life and depression in patients with an acute coronary syndrome: results from the RESPONSE randomised controlled trial

**DOI:** 10.1186/s12872-016-0321-4

**Published:** 2016-07-08

**Authors:** Harald T. Jørstad, Madelon Minneboo, Harold J. M. Helmes, Nick D. Fagel, Wilma J. Scholte op Reimer, Jan G. P. Tijssen, Ron J. G. Peters

**Affiliations:** Department of Cardiology, Academic Medical Center, Meibergdreef 9, 1105 AZ Amsterdam, The Netherlands; Department of Cardiology, Catharina Ziekenhuis, Eindhoven, The Netherlands; Amsterdam School of Health Professions, Amsterdam, The Netherlands

**Keywords:** Health-related quality of life, Depressive symptoms, Nurse coordinated prevention programme, Acute coronary syndrome

## Abstract

**Background:**

Improvement of health-related quality of life (HRQOL) is an important goal in preventive cardiology. HRQOL is also related to depressive symptoms, which represent a common co-morbidity and risk factor in patients with an acute coronary syndrome (ACS). Comprehensive nurse-coordinated prevention programmes (NCPP) in secondary care have been shown to reduce cardiovascular risk, however their effects on HRQOL and depressive symptoms have not been evaluated. We therefore investigated HRQOL and depressive symptoms in a secondary analysis in the RESPONSE trial, evaluating the effect of a NCPP on cardiovascular risk.

**Methods:**

RESPONSE was a multicentre (*n* = 11) randomised controlled trial in ACS-patients in secondary and tertiary healthcare settings evaluating a NCPP. The intervention consisted of four outpatient nurse clinic visits in the first 6 months after the index event, focusing on healthy lifestyles, biometric risk factors and medication adherence, in addition to usual care. The control group received usual care only. The outcome was change in HRQOL as measured by the MacNew questionnaire and change in depressive symptoms as measured by Beck’s Depression Inventory (BDI) questionnaire at 12-months follow-up relative to baseline.

**Results:**

Of 754 patients randomised, 615 were analysed for HRQOL; 120 for depressive symptoms. At baseline, HRQOL was 5.17 (SD 1.09) and 5.20 (SD1.04) (scale range 1.0 to 7.0) in the intervention and control group, respectively. At 12 months follow-up, HRQOL increased by 0.57 (SD 0.89) in the intervention group as compared with 0.42 (SD 0.90) in the control group (*p* = 0.03). This increase was observed across all relevant subscales. The BDI decreased by 1.9 in the intervention group as compared with 0.03 in the control group (*p* = 0.03) (scale range 1.0 to 63).

**Conclusion:**

Participation in a NCPP is associated with a modest but statistically significant increase in HRQOL, and a decrease of depressive symptoms, both of which are highly relevant to patients. A reduction in depressive symptoms may in addition contribute to a reduction in the overall risk of recurrent events.

**Trial registration:**

Dutch trials register: NTR1290. Registered 24 April 2008.

## Background

Patients with established coronary artery disease are at high risk of recurrent coronary events and mortality. Effective secondary prevention, including optimal medical therapy and lifestyle interventions (i.e. smoking cessation, healthy diet, weight loss/maintenance and regular exercise) can significantly reduce this risk [1, 2]. Nurses acting as disease managers have been demonstrated to be effective in reducing cardiovascular risk in several prevention programmes [3–6]. Therefore the European guideline on cardiovascular disease prevention and the World Health Organisation recommend nurse-coordinated prevention programmes (NCPP) to be integrated into healthcare systems [2, 7]. Such programmes are increasingly being implemented in clinical practice. We have previously shown that participation in a NCPP as part of the RESPONSE (Randomised Evaluation of Secondary Prevention by Outpatient Nurse SpEcialist) trial leads to a reduction in cardiovascular risk and a reduction of hospital admissions. This trial included patients with an acute coronary syndrome (ACS); most of who had multiple risk factors, including a high prevalence of lifestyle related risk factors. In short, the intervention group received nurse-coordinated care on top of usual care, while the control group received usual care only. We found that a NCPP improves risk factor control after 1 year. However, lifestyle-related risk factors, such as smoking and overweight, remained largely unchanged, with the exception of physical activity, where an improvement was observed (albeit self-reported) [7].

Health related quality of life (HRQOL), including emotional, physical and social well-being, is an important goal in preventive cardiology, in addition to optimal risk factor control [8]. Patients with an unhealthy lifestyle have been shown to have a lower HRQOL [9]. Nevertheless, lifestyle interventions have been shown to improve HRQOL [10]. Furthermore, HRQOL is influenced by a wide range of factors, such as patient characteristics and emotions, but also by factors as the quality of information and communication, factors which are targeted by NCPPs.

Furthermore, HRQOL is related to depressive symptoms, and depression is a common comorbidity among ACS-patients, with an incidence in the year after an acute myocardial infarction ranging from 10 to 30 % per year [11–13]. Major and minor depressions have been shown to be independent risk factors for cardiovascular mortality [14, 15]. Recently the American Heart Association listed depression as a risk factor for adverse medical outcomes in ACS-patients [15]. Depression is also associated with a higher prevalence of unhealthy behaviour, such as smoking and a sedentary lifestyle, and depression per se may also contribute to poorer cardiovascular outcomes [16–18].

Participation in a NCPP may improve HRQOL or depressive symptoms, resulting from continued care and attention to the patient’s personal situation. However, as NCPPs usually focus on risk factors, there may potentially be a detrimental effect on HRQOL or depressive symptoms if the attempted lifestyle changes are unsuccessful or if the effort to change one’s lifestyle is too arduous. For individual patients, smoking cessation, weight loss and diets, and limitations in alcohol intake may be perceived as a limitation of HRQOL, while the preventive effects (if successful) are not directly experienced (i.e. reduction of risk) [19, 20]. Therefore it is important to investigate whether a NCPP impacts on HRQOL and on depressive symptoms in a groups of ACS-patients and a high prevalence of CVD risk factors, including lifestyle related risk factors. We therefore aimed to investigate the change in HRQOL and depressive symptoms in the RESPONSE-population.

## Methods

### Study design

The RESPONSE trial was a multicentre, randomised controlled trial conducted in 11 hospitals in the Netherlands from June 2006 to July 2009. The study was designed to evaluate the impact of a practical, hospital-based NCPP on top of usual care in patients hospitalised for an ACS. The RESPONSE trial has been described in detail elsewhere [7, 21], and is briefly summarised below. (http://www.trialregister.nl/trialreg/admin/rctview.asp?TC=1290).

### Patient population

Eligible patients were 18–80 years of age, admitted for ACS (ST-segment myocardial infarction, non-ST-segment elevation myocardial infarction or unstable angina pectoris) The window of inclusion was up to 8 weeks after the date of discharge ACS. Exclusion criteria were: 1) unable to visit outpatient clinic 2) not available for follow-up 3) surgery or percutaneous coronary intervention expected within 8 weeks after inclusion 4) limited life expectancy (≤2 years) 5) previously enrolled in a NCPP, 6) congestive heart failure New York Heart Association class III or IV.

Patients were screened during or shortly after hospitalisation by their treating physician or a trained nurse. Patients were randomised using an online randomisation protocol. The online randomisation protocol consisted of a pre-generated block-stratified randomisation protocol (www.responsestudie.nl). Study personnel entered patients’ initials, date of birth and gender, and participating individuals were assigned a study identification number along with their allocation to either the intervention or control group. The randomisation protocol was designed and generated by an independent third party; study personnel had no influence on the randomisation process [7, 21]. The Academic Medical Center Ethical Review Board and institutional committees on human research of all recruiting hospitals approved the protocol and informed consent was obtained from all patients.

### Intervention group

Patients randomised to the intervention group were invited to attend the NCPP in addition to usual care. This programme included 4 outpatient clinic visits to a cardiovascular nurse during the first 6 months after inclusion. Between 6 months and 1 year there were no visits to the NCPP.

The NCPP was developed based on national and international guidelines, focusing on (1) healthy lifestyles, (2) biometric risk factors, and (3) medication adherence [1, 2, 22]. Each visit was structured by pre-defined topics, including smoking status, dietary status, level of physical activity, and medication use. Smoking was defined as smoking prior to the index event, physically inactive was defined as less than 30 min of moderate physical activity per day for at least 5 days per week [2]. The nurse provided advice on lifestyle and gave individual counselling and education as appropriate. During each visit weight, waist circumference, blood pressure, lipid profile (total cholesterol, LDL-cholesterol, HDL-cholesterol, triglyceride), fasting glucose and HbA1c were measured. For each variable, a target value was defined. When this target value was not reached, medication could be changed (in collaboration with the treating physician), or the patient could be referred to another health professional, in addition to counselling and advice.

Study nurses were all registered nurses with a 4-year bachelor’s degree, and competent in cardiac care. As part of the study, nurses were trained during a 3 day course in the principles of motivational interviewing, a method often utilised to achieve lifestyle changes [23].

### Control group

Patients randomised to the control group received usual care only, including visits to their treating cardiologists and other relevant specialists, and were offered cardiovascular rehabilitation according to national guidelines [22].

### Data collection

For our analysis, we used data collected at baseline and 12 months follow-up. Demographics (gender, educational status, work status, civil status and ethnicity), and cardiovascular risk factors (cardiovascular history, smoking status prior to index event, dietary status, level of physical activity and medication) were self-reported. Weight, height, waist circumferences, and blood pressure were objectively measured. Fasting blood samples were analysed for lipid profiles, glucose and HbA1c.

### Health-related Quality of Life (HRQOL)

We used the MacNew Heart Disease Heart-related Quality of Life questionnaire (MacNew) to measure quality of life. MacNew is a self-administered instrument consisting 27 items related to three domains of HRQOL: emotional, physical and social quality of life. Each item is rated on a 7–point Likert scale, where ‘1’ indicates poor HRQOL and ‘7’ indicates good HRQOL. A total score is calculated by taking the average of the score on each item. Missing items do not contribute to the total score, and if more than 4 items were missing a total score is not calculated. An emotional subscale score is calculated by 14 items (questions 1, 2, 3, 4, 5, 6, 7, 8, 10, 15 and 18), a physical subscale score by 13 items (questions 2, 6, 9, 11, 14, 16, 17, 19, 20 and 21), and a social subscale score by 13 items (questions 11, 12, 13, 15, 22, 23 and 27). The MacNew has been shown to be a valid, reliable and responsive questionnaire for patients diagnosed with myocardial infarction and angina pectoris [24].

### Depression screening

Data collection on depressive symptoms was added in a subset of patients (included in 6 hospitals) after initiation of the main study from September 2008 till July 2009 (protocol addendum). For depression screening, we used the Beck Depression Inventory (BDI). The BDI is a 21 item self-report questionnaire, developed to assess the presence and severity of depressive symptoms. Each item is rated on a 0–3 scale. A total score is presented as the sum of all items. The BDI is a reliable and validated measure of depressive symptomatology [25]. A BDI score ≥ 10 indicates at least mild to moderate symptoms of depression and has been associated with poor prognosis in patients with myocardial infarction [14]. For our analysis, patients with a BDI score higher or equal to 10 were classified as depressed, and patients with a BDI score lower than 10 were classified as non-depressed.

### Study outcomes

The impact of the NCPP on HRQOL was measured as the change in mean score of the MacNew questionnaire between baseline and 12 months, comparing the intervention group with the control group. The impact of the NCPP on depressive symptoms was similarly measured in a subgroup using the BDI. Both outcomes were secondary outcomes of the RESPONSE trial.

### Statistical methods

Continuous variables with a normal distribution were presented as mean and standard deviation (SD); categorical variables were presented as a number and percentage. Comparisons between groups for continuous data were analysed by independent samples t-tests or Mann Whitney U-tests, categorical data by χ2 tests or Fisher’s exact tests, as appropriate. The effect of time and group on the Macnew score was analysed by an analysis of variance (ANOVA) with repeated measures. SPSS statistics version 22.0 was used for all statistical analyses.

## Results

**Fig. 1:**
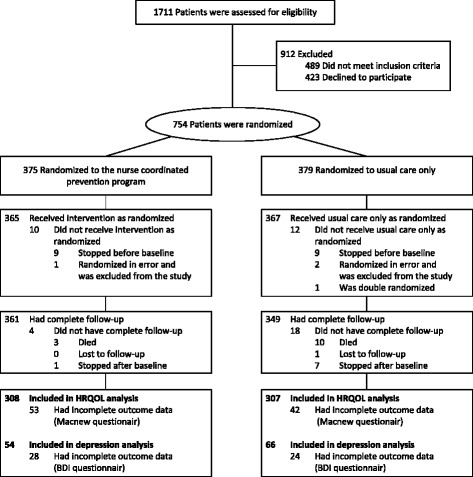
Study flowchart

A total of 1711 patients were screened for eligibility, and 754 patients were included and randomised in the RESPONSE study; of those 710 (94 %) patients attended 12 months follow up. For the present analyses, we included 615 (87 %) patients with complete MacNew questionnaires at baseline and 12 months (308 in the intervention group and 307 in the control group) (Fig. 1). Patients who did not have complete questionnaires were younger, less educated, unmarried or single, and had less peripheral artery disease. Of these 615 patients, 120 (20 %) patients had complete BDI-data at baseline and 12 months.

Patient characteristics are presented in Table 1. The mean age was 58 years, and 20 % of the patients were female. The majority (73 %) of the study population had no known previous cardiovascular disease before admission for an ACS. In total, 44 % of the patients were current smokers, more than 70 % had a BMI > 25 kg/m2, and 49 % were physically inactive. Characteristics of the patients screened for depressive symptoms were comparable with the total group. At baseline the BDI score was 8.1 (SD 7.2) in the intervention group and 6.1 (SD 5.1) in the control group. Fifteen (28 %) patients were depressed in the intervention group and 14 (21 %) patients were depressed in the control group (BDI >10). There were no significant differences in baseline characteristics between the two groups.

**Table 1:** Baseline characteristics

	Included in HRQOL analysis				Included in depression analysis			
	Intervention		Control		Intervention		Control	
	*n* = 308		*n* = 307		*n* = 54		*n* = 66	
Demographics								
Age (years)	57.7	(9.5)	58.2	(9.7)	57.3	(9.3)	58.1	(10.0)
Female	63	(21 %)	63	(21 %)	12	(22 %)	15	(23 %)
Caucasian	282	(92 %)	281	(92 %)	49	(91 %)	62	(94 %)
Higher education (>8 years)	69	(22 %)	65	(21 %)	11	(20 %)	12	(18 %)
Employed (fulltime or part-time)	162	(53 %)	171	(56 %)	32	(60 %)	37	(56 %)
Married/cohabiting	250	(81 %)	257	(84 %)	42	(84 %)	57	(88 %)
Previous vascular disease								
Myocardial infarction	51	(17 %)	54	(18 %)	10	(19 %)	10	(15 %)
Percutaneous coronary intervention	41	(13 %)	46	(15 %)	6	(11 %)	6	(9 %)
Coronary artery bypass surgery	14	(5 %)	17	(6 %)	5	(9 %)	3	(5 %)
Stroke	12	(4 %)	7	(2 %)	0	(0 %)	0	(0 %)
Peripheral artery disease	18	(6 %)	22	(7 %)	6	(11 %)	7	(11 %)
No known previous cardiovascular disease	224	(73 %)	222	(73 %)	37	(69 %)	52	(80 %)
Cardiovascular risk factors								
Positive family history	179	(58 %)	187	(61 %)	30	(56 %)	40	(62 %)
Diagnosed diabetes mellitus	42	(14 %)	42	(14 %)	6	(11 %)	8	(12 %)
Dyslipidaemia before hospital admission	214	(70 %)	218	(71 %)	35	(65 %)	42	(64 %)
Hypertension before hospital admission	120	(39 %)	107	(35 %)	19	(35 %)	22	(33 %)
Current smoking	142	(46 %)	126	(41 %)	27	(50 %)	26	(39 %)
Overweight (BMI > 25)	241	(78 %)	219	(71 %)	44	(82 %)	48	(73 %)
Physically inactive	155	(50 %)	149	(49 %)	24	(44 %)	32	(49 %)
Depression parameters								
BDI score					8.1	(7.2)	6.1	(5.1)
BDI ≥ 10					15	(28 %)	14	(21 %)

Table 2 presents the scores and the changes in MacNew between baseline and 12 months follow-up. HRQOL improved in both groups. There was a slight but statistically significant greater improvement in MacNew scores at 12 months in favour of the intervention group [Intervention +0.57 (SD 0.89) vs. control +0.42 (SD 0.90) *p* = 0.03]. This improvement was consistent across all three dimensions of the questionnaire (emotional, physical and social). The absolute difference in mean change between the intervention and control group was 0.15 (95 % CI 0.02–0.29 *p* = 0.03). No intervention effect was seen with repeated measurements ANOVA (*p* = 0.55), although the effects of time and the interaction between time and group, were significant (*p* < 0.001 and *p* = 0.03, respectively).

**Table 2:** Change in MacNew score (HRQOL) from baseline to follow up

	Baseline				12 months				Change from baseline to 12 months						
	Intervention		Control		Intervention		Control		Intervention	Control					
	*n* = 308		*n* = 307		*n* = 308		*n* = 307		*n* = 308	*n* = 307	Mean difference	St. Error of the Mean	95 % C.I.		
	Mean	(SD)	Mean	(SD)	Mean	(SD)	Mean	(SD)	Mean	Mean			Lower	Upper	*p*-value
MacNew total	5.17	(1.09)	5.20	(1.04)	5.74	(0.94)	5.62	(1.07)	0.57	0.42	0.15	0.07	0.02	0.29	0.03
Emotional subscale	5.04	(1.22)	5.03	(1.15)	5.56	(1.05)	5.40	(1.12)	0.51	0.37	0.15	0.08	−0.12	0.30	0.07
Physical subscale	5.01	(1.19)	5.07	(1.15)	5.65	(1.08)	5.54	(1.18)	0.64	0.46	0.18	0.08	0.02	0.34	0.03
Social subscale	5.51	(1.15)	5.53	(1.11)	6.16	(0.93)	6.03	(1.05)	0.64	0.49	0.15	0.08	−0.01	0.31	0.06

In patients with depression screening, the intervention group showed a decrease of 1.9 points as compared with 0.03 points in the control group (*p* = 0.03). The mean difference between the intervention and the control group was −1.84 points [(95 % C.I. -3.45 to −0.20) *p* = 0.03]. At 12 months, 12 patients in the intervention group were depressed as compared to 11 in the control group (*p* = ns).

## Discussion

The main finding of our study is that participation in a NCPP leads to an increase in HRQOL on top of improved risk factor control in patients who have been hospitalised for an ACS. This increase in HRQOL was seen across all the emotional, physical and socials subscales. Furthermore, a NCPP contributes to a reduction in depressive symptoms in ACS patients. However, there was no difference in the number of depressed patients between the intervention and control group based on a binary definition.

In addition to morbidity and mortality outcomes, HRQOL plays an important role in treatment strategies. A consensus statement from the Society for Cardiovascular Angiography and Interventions advocates that HRQOL outcomes should be measured in clinical trials and guidelines [26]. In line with this, our study reports the HRQOL outcomes from a large, randomised controlled trial investigating the effect of such a programme on cardiovascular risk factors.

Several studies in patients with coronary artery disease have shown that HRQOL improves after treatment in patients with coronary artery disease undergoing a percutaneous coronary intervention, coronary artery bypass grafting or treated with optimal medical therapy [27–29]. In our study, revascularisation rates in both groups were comparable. Patients randomised to the intervention group received greater emphasis on improvement of risk profiles through adherence to medication and changing unhealthy lifestyles. Usual care, as provided in both groups, included visits to cardiologists, general practitioners and other health care personnel, and there were no restrictions in either group as to participation in cardiac rehabilitation programmes. Accordingly, the observed improvement in HRQOL was achieved against a background of a high level of usual care, and with excellent adherence to medication in both groups [21].

Clinically meaningful changes in the total score of the MacNew have been reported to be in the magnitude of 0.5, similar to our results [30]. Although both groups showed improvement in quality of life, the intervention group improved more than the control group. Potential explanations for this improvement include more individual attention from the nurses, and their ability to respond to individual patient’s needs, as well as providing better information.

In the EuroAspire III survey (2006–2007), conducted in 8745 patients with coronary artery disease in 22 European countries, HRQOL was shown to be higher in patients adopting healthier lifestyles [31]. However, the EuroAspire III survey was designed as a cross-sectional study, and the direction of the association between HRQOL and lifestyle is uncertain. Adding to their observations, our study shows prospectively that attending an NCPP (on top of usual care) improves both medication adherence and lifestyle components and leads to a greater improvement in HRQOL than usual care alone.

Murchie et al. (2004) investigated the effect of a NCPP on quality of life (QOL) as measured by Short-Form 36 (SF-36). At 12-months follow-up, they showed a significant improvement in 5 of 8 domains of SF-36, comparable with our findings [32]. However, our NCPP took place in a hospital setting and, by comparison, we included younger patients (58 years vs 66 years) with a more recent coronary event. Furthermore, our measure of interest was HRQOL as opposed to generic QOL. In a cluster-randomised trial in primary practices, Khunti et al. (2007) found a slight increase in QOL (SF36) in patients with coronary heart disease attending specialist nurse managers as compared with usual care [33]. This increase reached statistical significance in the domains of physical functioning, general health, vitality, social functioning and mental health. This study population consisted mainly of chronic patients, and the intervention was delivered in the primary practice.

Consistent with our findings, a meta-analysis by Ekers et al. showed that nurse-delivered collaborative care is an effective treatment for depression in patients with different chronic health problems, including in coronary heart disease. However, studies were included with nurse interventions specifically targeting depression, where nurses received brief periods of training for this purpose [34]. In our study, nurses received training in motivational interviewing and secondary prevention, but not specifically for depression treatment, as depression treatment was not a pre-specified target in our study. Possibly, the nurse and the extra attention and care received by patients visiting a NCPP have, on their own, a modestly positive effect on depressive symptoms. Furthermore, while the mean decrease in BDI was greater in the intervention group as compared with the control group, this did not change the prevalence of the number of depressed patients at 12 months, with 12 (22 %) patients in the intervention group and 11 (17 %) patients in the control group having a BDI > 10 after 1 year.

### Strengths and limitations

There are several strengths to our study. First, we assessed HRQOL in a large, contemporary randomised multicentre trial with a well-defined trial population. Second, the NCPP investigated in the trial was a practical intervention with clearly defined intervention components, facilitating future implementation of comparable programmes. Finally, complete questionnaires on HRQOL were available in the majority of patients, making selection bias unlikely.

Some limitations should be considered when interpreting our results. First, we did not collect data on systolic left ventricular (dys) function. However, no patients in our trial had severe, symptomatic heart failure, as we excluded all patients with heart failure NYHA class III or IV. Only 6 patients (in the control group) were hospitalised for congestive heart failure during follow-up, making congestive heart failure unlikely as a cause for the slight difference in HRQOL observed between the groups [7]. Second, we excluded patients >80 years of age. While secondary prevention should always be considered in the context of life expectancy, improvements in HRQOL would likely be of value in elderly and frail patients. While it is conceivable that an increase in HRQOL may be observed in patients older than included in our trial, we cannot infer this from our data. Additional factors, such comorbidity and frailty should be taken into account when evaluating comparable interventions in these populations [35, 36]. Third, our trial included a slightly lower proportion of women as compared with other national and international surveys. In the European Action on Secondary and Primary Prevention by Intervention to Reduce Events (EUROASPIRE) III survey, performed in 22 countries in Europe (including the Netherlands), 27 % of participants were women [37]. Fourth, we collected data about depression in a small sample, as this component of the study was added to the protocol after initiation of the main trial. Although only a modest number of patients were screened for depressive symptoms, we observed a small but significant difference between the groups.

## Conclusion

Our study suggests that participation in a nurse-coordinated prevention programme with four outpatient clinic visits in addition to usual care leads to a small but significant improvement in HRQOL in patients with an ACS. This improvement was seen across the emotional, physical and social dimensions of HRQOL. In addition to the effect on HRQOL, the NCPP was found to reduce depressive symptoms in these patients. In conclusion, our study shows that a NCPP has a favourable effect on HRQOL and depressive symptoms in patients who have been hospitalised for an ACS.

## Abbreviations

ACS, acute coronary syndrome; BDI, Beck’s Depression Inventory; HRQOL, Health-related quality of life; NCPP, nurse-coordinated prevention programmes; QoL, quality of life; RESPONSE, Randomised Evaluation of Secondary Prevention by Outpatient Nurse SpEcialist; SD, standard deviation; SF-36, Short-Form 36.
